# Counseling clients during New York City’s COVID-19 pandemic: observations on fundamental elements of emotions management

**DOI:** 10.1017/ipm.2020.42

**Published:** 2020-05-14

**Authors:** M. Sweeney

**Affiliations:** Metropolitan Center for Cognitive Behavioral Therapy, New York, USA

**Keywords:** coping, COVID-19, New York City, pandemic

## Abstract

New York City is in the grip of the COVID-19 pandemic. Health care centers are stretched beyond capacity. Daily death rates are staggering. The city’s population is hunkered down in fear. Our anxiety treatment center is treating patients via video appointments. We are helping anxious individuals adapt to tumultuous changes that we ourselves are experiencing. Our work in this time has reinforced our core beliefs about managing one’s emotions; that difficult times require more active coping and that we all draw heavily from social support and familiarity to create a feeling of well-being. These principles and the experiences of our patients are discussed.

The city that never sleeps is shuttered. Fifth Avenue, the route of countless parades, is desolate. The density of life that lends New York City its vigor is also contributing to the speed with which COVID-19 has spread through the city’s population. The vacant streets are largely the result of sensible people participating in social distancing, standing together by staying apart. Social distancing is necessary to flatten the curve but is also significantly contributing to psychological distress. Below I outline some of the psychological effects of physical distancing, our clinical observations and recommendations, and some of my personal experiences.

At the time of this writing, NYC has in excess of 160,000 identified cases, 40,000 hospitalized individuals many in ICU units, and over 18,000 deaths. An additional untold number of people are ill at home. The doctors, nurses, and other frontline medical staff have proven themselves to be heroes. Individuals, grief stricken by the loss of a loved one, must mourn in an odd semi-isolation stripped of many of the usual cultural rituals surrounding death. The trauma of the frontline staff and the grief of those who lost a loved one require specific and dedicated psychological attention. Far less acute, but far more common, are the psychological effects of social distancing.

The psychological effects of social distancing are highlighting important truths; among them, that we rely on one another for emotional support and that we are buoyed by the feeling of familiarity. We are all makers of our own mood, but what we make relies on the ingredients we have. Social distancing has limited our access to the ingredients needed for the feeling of well-being. A partial list of ingredients includes structure, predictability, a sense of agency and accomplishment, sleep, exercise, and social support. Additionally, a feeling of well-being is facilitated by employing emotion management skills, including planning, perspective taking, the capacity for delay, and the ability to challenge one’s darker thoughts. I will highlight two significant contributors to the feeling of well-being: familiarity and social support.

Familiarity may have been the first tent pole of emotional well-being to fall. Thrown into a global pandemic fraught with unknowns, our sense of familiarity is displaced. One’s attention is drawn to the news, the awful events, and speculation about an unwanted new normal. Familiarity is an important contributor to the feeling of well-being; it enhances the feeling of competency. Familiarity gives one the psychological equivalent of feeling as if your feet are on the floor. The familiarity with which one completes the tasks of daily life provides a comforting feeling; I know this, I have seen this before, I know what will come next, there is no cause for alarm, nothing will be required of me that that I have not previously been able to do. There is little ‘been there done that’ in our current situation. Engagement with the ordinary makes time pass easily; it increases one’s feeling of certainty and predictability. Our clinical observation is a strong association between drift from ordinary routine and increased distress. Some people are finding it hard to make and maintain routines and structure time without the external structure of work, school, and commute. At home, the hour of the day can hold less meaning, a lifestyle separated from the clock and calendar diminishes the feelings of structure, predictability, and purpose. Consequently, the advice to our patients is to enhance the feeling of familiarity. To create new routines even if they must be restricted to one’s home. To keep a regular time of waking up and going to sleep. To see and speak with many of the same people. We encourage people to stick to their ordinary routines for sleep, work hours, exercise, alcohol consumption, and social connectedness, etc. The new routine need not be identical to the former routine, but a routine is necessary to enhance the feeling of familiarity. Personally, some of my easiest days during this crisis are days when I am at work, albeit by teletherapy. Work bestows upon me a wonderful feeling of familiar and purposeful. I can involve myself in the lives of others and be pulled out of my own concerns.

Social support may be the single most potent contributor to emotional well-being. Social support provides numerous benefits: a source of distraction, a sense of connection, an involvement outside of one’s self, a sounding board for ones worries, and a source of joy, among other benefits. As is said, a joy shared is doubled and a troubled shared is halved. Social distancing challenges the availability and context of social support. A good deal of social support comes as a by-product of our daily routine. The small bits of kind incidental contact with individuals with whom you might not otherwise interact. The casual conversation with a coworker, a teacher, or a store clerk provides a small but meaningful boost to one’s mood. It is a net positive that comes with little effort. That easily provided socializing ends when one’s daily trips are curtailed. Social support from close friends continues but is changed in significant ways; physical proximity is diminished, ones support sources are also stressed, and all conversations focus on COVID-19. Physical proximity adds to the feeling that one is with you; the emotional benefits of a pat on the back or a hand your shoulder is hard to quantify but certainly noticed when missing. Similarly stressed, discussing your feelings with a friend not caught up in the same situation gives one an independent calm voice who might offer a different perspective and dilute your worry. When all the individuals are similarly stressed the diluting feature of support is diminished. Interactions that once covered a variety of topics are now dominated by the single gruesome topic of daily pandemic news. The net effect is that social support as a tool for psychological well-being can lose some of its mood enhancement luster when all conversations remind you of the sad truth of the day. The need for social connection is exemplified by the city’s newest tradition; at 7 pm each evening, the time of the hospital workers shift change, the entire city pauses to clap and cheer for the heroes who are keeping us safe. Some individuals have been spotted playing the trumpet. Our clinical observation is a strong association between diminished social contact and increased distress. Consequently, we are problem solving with patients on methods to keep a high quality of social connectedness. Please connect with others. Please see how vital they are for your well-being and how vital you are for theirs. Try in these interactions to cover the widest variety of topics. Make sure that coronavirus and its effects are not the only thing that you are discussing. Personally, I have been at my best when I am most engaged with family and friends. I have made a conscious attempt to focus my non-work time on non-Covid topics. Silly, productive, engaging, and positive future focused conversations have been a great reprieve after a long day of client calls.

In conclusion, it is a dark and extraordinary difficult time in NYC. I am humbled by the selfless dedication of hospital staff. I am saddened for those who are lost and who have lost loved ones. Coping with COVID-19 is a job for each of us. There is no merit badge for being stressed in stressful situations. Difficult times require extra effort to manage one’s emotions. Additionally, the work of emotions management is harder because some of the common ingredients for emotional well-being are harder to access during social distancing. Personally, I am happy when I can contribute in my own small way. I am happy for my work; it provides the familiarity, social connection, and sense of purpose that I rely on for my own emotional well-being.

